# Secondary metabolites of *Bacillus subtilis* L2 show antiviral activity against pseudorabies virus

**DOI:** 10.3389/fmicb.2023.1277782

**Published:** 2023-10-30

**Authors:** Xiaoli Wang, Guijuan Hao, Meng Zhou, Meng Chen, Hongli Ling, Yingli Shang

**Affiliations:** ^1^Department of Preventive Veterinary Medicine, College of Veterinary Medicine, Shandong Agricultural University, Taian, China; ^2^Shandong Provincial Key Laboratory of Animal Biotechnology and Disease Control and Prevention, Shandong Agricultural University, Taian, China; ^3^Qingdao Vland Biotech Inc., Qingdao, China; ^4^Institute of Immunology, Shandong Agricultural University, Taian, China

**Keywords:** *Bacillus subtilis*, secondary metabolites, pseudorabies virus, antiviral activity, probiotics

## Abstract

*Bacillus subtilis* (*B. subtilis*) is a commercially important probiotic known to produce secondary metabolites with antibacterial, antifungal and anti-inflammatory activities. However, the potential ability of *B. subtilis* to combat viruses, especially DNA viruses, has not been extensively investigated. In this study, we identified two distinct *B. subtilis* strains and examined the efficiency of their secondary metabolites against pseudorabies virus (PRV), a swine herpesvirus resulting in economic losses worldwide. We found that treatment with the secondary metabolites of *B. subtilis* L2, but not the metabolites of *B. subtilis* V11, significantly inhibited PRV replication in multiple cells. Notably, the antiviral activity of the metabolites of *B. subtilis* L2 was thermal stable, resistant to protease digestion. Moreover, these metabolites effectively impeded PRV binding, entry and replication. Importantly, oral administration of the metabolites of *B. subtilis* L2 protected mice from lethal PRV infection, rescuing weight loss and reducing the viral load *in vivo*. In summary, our results reveal that the metabolites of *B. subtilis* L2 exhibit anti-PRV activity both *in vitro* and *in vivo*, providing a potential candidate for novel antiviral drugs.

## Introduction

Probiotics, currently defined as microorganisms associated with beneficial effects for humans and animal health, are gaining much interest as alternatives for antibiotics or anti-inflammatory drugs (Zolkiewicz et al., 2020). In addition to various types of food products, probiotics are widely used in therapies to multiple diseases including suppression of diarrhea, alleviation of lactose intolerance and postoperative complications (Bermudez-Brito et al., 2012; Kassaa, 2017). It has been shown that several species belonging to the genera including *Bacillus*, *Lactobacillus*, and *Enterococcus* can improve animal production and health as animal feed additives (Chaucheyras-Durand and Durand, 2010). Interestingly, recent studies show that probiotics or its metabolites also exhibit antiviral activity on human viruses (Kassaa, 2017; Wang et al., 2022). For example, oral administration of *Lactobacillus gasseri* SBT2055 effectively controlled respiratory syncytial virus infection in mice by reducing lung viral loads and pro-inflammatory cytokines as well as enhancing expression of interferons (Eguchi et al., 2019). Moreover, the metabolites released by *Lactobacillus*, such as H_2_O_2_ and lactic acid also have antiviral effects on human immunodeficiency virus (O’Hanlon et al., 2011; Gosmann et al., 2017; Tachedjian et al., 2017). However, the antiviral effect of probiotics against animal viruses still remains obscure.

*Bacillus subtilis* (*B. subtilis*) has long been used as a probiotic for the treatment of intestinal diseases in humans and animals, and in recent decades has been developed for use as animal feed enhancers and antifungal biocontrol agents (Joshi et al., 2008; Harwood et al., 2018; Rhayat et al., 2019). *B. subtilis* can also produce a range of secondary metabolites, including polyketides, terpenes and siderophores, or ribosomally and non-ribosomally synthesized peptides to exert their inhibitory effect on bacterial pathogens *in vitro* and *in vivo* (Nannan et al., 2021; Ji et al., 2022; Mazhar et al., 2022; Qiu et al., 2022). Recently, it was shown that the secondary metabolites of *B. subtilis* has antiviral activity. For instance, surfactin, a cyclic lipopeptide antibiotic and biosurfactant produced by *B. subtilis*, can act as a membrane fusion inhibitor with activity against enveloped RNA viruses including transmissible gastroenteritis virus, thereby inhibiting viruses from entering the intestinal epithelial cells (Yuan et al., 2018, 2019). In addition, a peptide generated by *B. subtilis* 3 could completely inhibit the influenza virus *in vitro*, which was comparable to that of oseltamivir phosphate (Starosila et al., 2017). However, it remains unclear whether the secondary metabolites of *B. subtilis* could prevent viral infections caused by DNA viruses.

Herein, we identified two strains of *B. subtilis* L2 and V11 from broiler chicken fecal samples and evaluated their antiviral ability against pseudorabies virus (PRV), a canonical DNA virus belongs to herpesvirus that causes severe disease in swine. We found that the metabolites of *B. subtilis* L2 and V11 exhibited distinct biological activities. Specially, *B. subtilis* L2 demonstrated significant inhibition and prevention of PRV replication in PK-15 cells. Moreover, oral administration of the metabolites of *B. subtilis* L2 also protected mice from lethal PRV infection and reduced viral load in the brain and lung. Thus, our findings reveal the potential ability of the metabolites of *B. subtilis* to combat the PRV infection and broaden the antimicrobial spectrum of *B. subtilis* metabolites.

## Materials and methods

### Isolation and identification of *B. subtilis* strains

*Bacillus subtilis* strains were isolated from broiler chicken fecal samples by regular Luria-Bertani (LB) agar medium as described previously (Rahimi et al., 2018) and were then identified by a MALDI Biotyper system (Bruker, Germany) as described elsewhere (Sehl and Teifke, 2020). Briefly, bacterial samples were prepared by the direct colony transfer procedure by the manufacturer’s extraction protocol. The RiboPrinter microbial characterization system was used for bacterial identification with Bruker standard reference database versions V.3.1.2.0 (3,995 entries) to V.9 (8,468 entries). A species cutoff score value of 2.0 and a genus cutoff score value of 1.7 were applied.

### Preparation and extraction of the secondary metabolites of *B. subtilis* (SMBS)

To obtain the SMBS, a single colony of *B. subtilis* L2 or V11 was inoculated into a test tube containing LB medium, then shaken at 37°C, 180 r/min for 24 h. The seed liquid was then inoculated to LB medium at a ratio of 1:10 for 24 h. Cultures were centrifuged at 10,000 *g* for 20 min at 4°C to remove solid bacteria. The supernatant containing SMBS was then collected, filtered with a 0.22 μm membrane and stored at −20°C for use. For isolation of the key components from SMBS-L2, acetone extracts were subjected to chromatography using a silica gel column. Eluent samples were collected with a stepwise gradient of ethyl acetate-methanol and were identified by blotting onto thin layer chromatography (TLC) plates to combine identical components. Samples were then evaporated, freeze-dried, and redissolved in distilled water for subsequent experiments.

### Viruses and cells

PK-15 cells, HeLa cells, and baby hamster kidney-21 (BHK-21) cell line were obtained from the American Type Culture Collection (ATCC, VA, USA). Cells were cultured in Dulbecco’s minimal essential medium (DMEM) (Gibco, USA) supplemented with 10% fetal bovine serum (FBS) (Biological Industries, Israel) and penicillin-streptomycin (Gibco) at 37°C in a CO_2_ incubator. The cells were regularly tested for mycoplasma contamination. The PRV Bartha-K61 strain was purchased from China Veterinary Culture Collection Center (Cat#CVCC AV249, Beijing, China) and was purified in BHK-21 cells. The green fluorescent protein (GFP)-tagged PRV (PRV-GFP) recombinant virus was generated previously (Liu et al., 2022).

### Cell viability assay

MTT [3-(4,5-dimethylthiazol-2yl)-2,5-diphenyl] (Sigma, USA) assay was used to examine the effect of SMBS on cell viability as described previously with minor modifications (Kumar et al., 2018). Briefly, PK-15 cells were cultured in a 96-well plate (10^4^ cells/well) for overnight and were then treated with SMBS at various concentrations as indicated. After 72 h, cells were washed twice with phosphate buffer saline (PBS) and incubated with 20 μL of MTT (5 mg/mL) for 4 h. The salt formed was solubilized by adding DMSO (150 μL/well) and shaking for 10 min. The optical density (OD) value was determined using an EnSpire Multimode Plate Reader (Thermo Fisher, USA) with a 540-nm excitation filter. Relative cell viability was then calculated as (mean OD_540_ of treated cells)/(mean OD_540_ of control cells) × 100%.

### Cell treatment, virus infection and flow cytometry

For analysis of SMBS on PRV infection, PK-15 cells, BHK-21 cells or HeLa cells were treated with SMBS at indicated doses following infection by PRV-GFP (MOI = 0.1, 0.5 or 1, respectively) for 24 h. Cells were then analyzed on an LSR Fortessa flow cytometer using FlowJo software (Version 10, BD Biosciences, USA) described previously with minor modifications (Zhang et al., 2021). For SMBS stability analysis, SMBS were treated at 60°C, room temperature (RT) or −20°C for 10 days, or were, respectively, treated with pepsin (Solarbio, China) in 0.02 N HCl at pH 4.0 or trypsin (Sigma, USA) with a final concentration of 100 μg/mL in 10 mM phosphate buffer (pH 7.0) at 37°C for 2 h before application. For specific analysis of SMBS on PRV binding, PK-15 cells were kept at 4°C for 1 h (precooled cells) following infection of PRV-GFP (MOI = 1, the same below) and SMBS treatment for another hour simultaneously, or PRV-GFP pretreated with SMBS at 4°C for 1 h before virus binding assay. Cells were then washed thrice with PBS (pH 7.0) following incubation in fresh DMEM for 24 h at 37°C before analysis. For specific analysis of SMBS on PRV entry, precooled PK-15 cells were infected by PRV-GFP at 4°C for an hour and were then washed thrice with PBS (pH 7.0) following culture in fresh DMEM containing SMBS at 37°C for another hour. Cells were then washed with PBS (pH 3.0) following incubation in fresh DMEM for 24 h at 37°C before further analysis. For specific analysis of SMBS on PRV replication step, PK-15 cells were infected with PRV-GFP at 37°C for 1 h and extracellular viruses were then washed out with PBS (pH 7.0). Cells were then incubated in DMEM containing SMBS for 24 h at 37°C before analysis.

### DNA extraction and quantitative polymerase chain reaction (qPCR)

Total DNA was extracted using a DNA isolation kit (Cat#DP304, TIANGEN, China) according to the manufacturer’s instructions. PRV copy numbers were quantified by qPCR using gD gene primers (Cheng et al., 2020) and was performed in triplicated determinants with RealStar Green Fast Mixture (A303, GenStar, USA) on a StepOne plus thermal cycler (ABI, Thermo Fisher Scientific, USA). The primer sequences targeted for the gD gene of PRV were followed as previously described (Zhao et al., 2022).

### Immunoblotting analysis

Immunoblotting analysis was performed as described previously (Ning et al., 2019). For total protein extraction, indicated cells were washed with cold PBS and lysed on ice in a lysis buffer (50 mM Tris, 150 mM NaCl, 1% Triton X-100, 1% sodium deoxycholate, 1 mM Na_3_VO_4_, 1 mM EDTA, and 1 mM PMSF). Whole-cell lysates were then separated by sodium dodecyl sulfate polyacrylamide gel electrophoresis (SDS-PAGE) and were transferred to polyvinylidene difluoride membrane (Millipore, USA) using semi-dry transfer system (Bio-Rad, USA) for immunoblotting with specific antibodies. Antibodies against green fluorescent proteins (1:5,000, 66002-1-Ig) and β-actin (1:20,000, 66009-1-Ig) were purchased from Proteintech Group, Inc., China.

### Reverse transcription and quantitative real-time PCR

Total RNA was extracted from whole-cell lysates with a total RNA purification kit (Cat#LS1040, Promega, Beijing, China) and reversely transcribed to cDNA with M-MLV reverse transcriptase (Takara, Dalian, China). qPCR was performed in triplicated determinants with RealStar Green Fast Mixture (A303, GenStar, USA). Threshold cycle numbers were normalized to triplicated samples amplified with primers specific for glyceraldehyde-3-phosphate dehydrogenase (*GAPDH*). Primers for gene *IFNB1* were followed as previously described (Kong et al., 2022).

### Animal experiments

All animal protocols were reviewed and approved by the Shandong Agricultural University Animal Care and Use Committee (Approval Number: # SDAUA-2018-057) and were conducted in strict accordance with the Animal Ethics Procedures and Guidelines of the People’s Republic of China. Six-week-old C57BL/6 mice from Beijing Vital River Laboratory Animal Technology Co., Ltd., were used for all experiments. All mice were maintained in pathogen-free barrier facilities. For survival assay, mice were intraperitoneally inoculated with 0.2 ml of 0.5 × 10^6^ PFU of PRV (Bartha-K61 strain, *n* = 5 in each group) (Kong et al., 2022). For SMBS treatment *in vivo*, 50 μL of SMBS of *B. subtilis* L2 or V11 per mouse were orally administered for 5 consecutive days immediately after PRV infection. Mice given the same amount of PBS were set as a negative control. Mice were recorded for 15 days and all mice were weighed daily. The lungs and brains of mice were collected at 6 dpi for detection of viral load or hematoxylin and eosin (H&E) analysis.

### Histopathology

For histopathology analysis, tissue samples from mouse brain or lung were fixed overnight in 10% neutral-buffered formalin, trimmed, and embedded in paraffin. Sections were then cut into 5 μm and stained with H&E (Cheng et al., 2020). Images were captured using on an Olympus microscope (CX41RF) with imaging software (MiE V3.1). Histological scores were determined as described previously (Matute-Bello et al., 2011; Ma et al., 2019).

### Statistical analysis

*P*-values were calculated with a two-tailed paired or unpaired Student’s *t*-test by Prism GraphPad software (v8.0). *P*-values of <0.05 were considered significant. For mice survival assay, statistical significance was analyzed with the log-rank test.

## Results

### *B. subtilis* strains L2 and V11 show distinct characteristics

**FIGURE 1:**
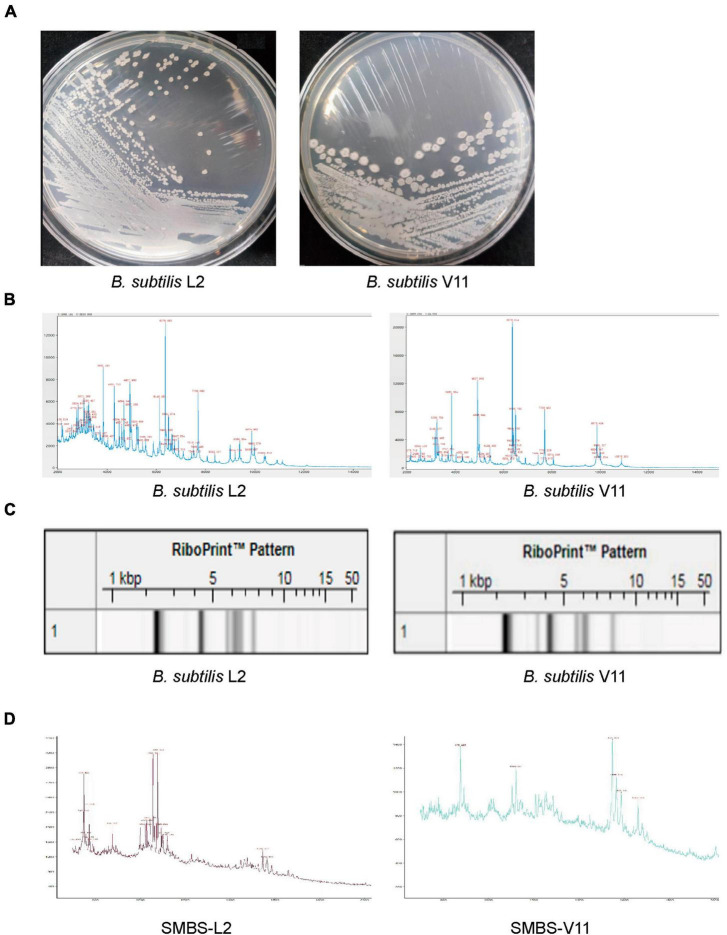
Identification of two novel *B. subtilis* strains L2 and V11. **(A)** The colony morphologies of *B. subtilis* L2 and V11 grown on LB agar plates after incubation at 37°C for 24 h. **(B)** The mass spectrum peaks of *B. subtilis* via MALDI-TOF MS analysis. **(C)** Genetic fingerprints of *B. subtilis* L2 and V11 generated by the RiboPrinter system. **(D)** MALDI-TOF MS analysis of the SMBS from L2 (SMBS-L2) and V11 (SMBS-V11) strains.

To obtain novel *B. subtilis* strains, we performed probiotic isolation from broiler chicken fecal samples. Two bacterial strains, named L2 and V11, were isolated and both strains exhibited cream opaque colonies on LB agar medium (Figure 1A). While the gross morphology of strain L2 colonies was small, smooth with well-defined edges, the morphology of strain V11 colonies displayed rough colony surfaces with elevated edges (Figure 1A), indicating that the isolated bacterial strains L2 and V11 are likely two different strains. To further identify the isolated strains, matrix-assisted laser desorption/ionization time of flight mass spectrometry (MALDI-TOF MS) was used for a rapid identification. MALDI-TOF MS analysis revealed that both strains belong to *B. subtilis* as shown by the key mass spectrum peaks (Figure 1B). In line with this observation, the RiboPrinter automatic strain identification system also showed that genetic fingerprints of the two strains matched to those of *B. subtilis* in the DuPont Free database (Figure 1C). In addition, MALDI-TOF MS analysis of the SMBS of L2 and V11 strains revealed that SMBS-L2 have more high-mass spectrum peaks between 700 and 1,200 compared to SMBS-V11, suggesting the presence of more small molecules in SMBS-L2 than that in SMBS-V11 (Figure 1D). Together, these data suggest that the newly identified probiotic strains L2 and V11 are *B. subtilis* with distinct characteristics.

### The secondary metabolites of *B. subtilis* L2 inhibit PRV infection *in vitro*

**FIGURE 2:**
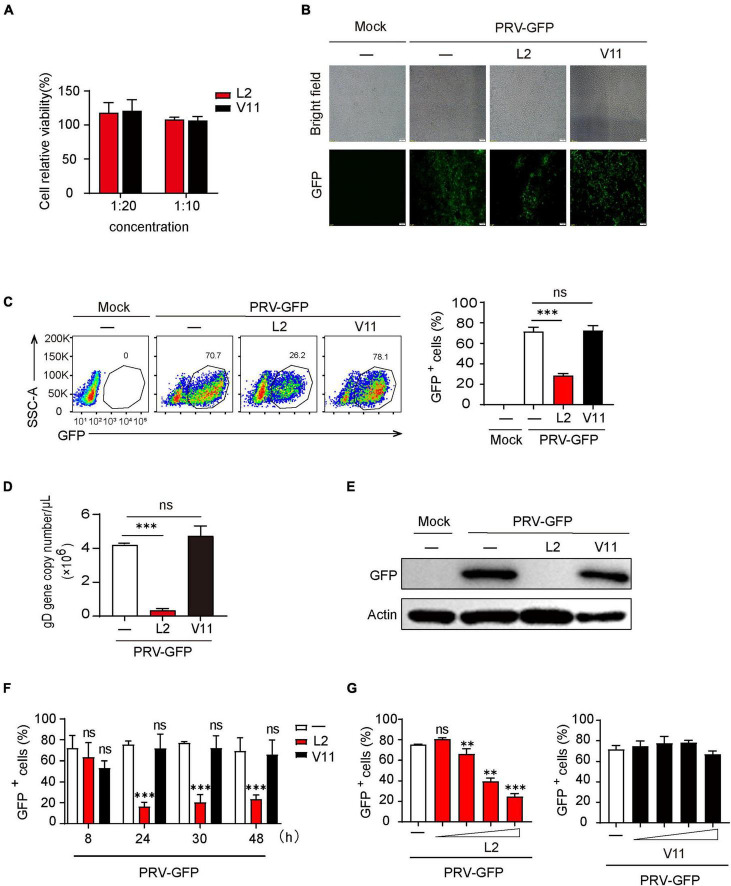
SMBS of L2 significantly inhibited PRV replication *in vitro*. **(A)** Cytotoxicity analysis of SMBS on PK-15 cells by MTT assay at indicated test concentrations. **(B,C)** Fluorescence microscope **(B)** or flow cytometry analysis **(C)** of PK-15 cells infected with PRV-GFP (MOI = 0.1, the same below) following treatment with or without SMBS. **(D)** qPCR analysis of glycoprotein D (gD) gene copy number in PK-15 cells infected with PRV-GFP. **(E)** Immunoblotting analysis of GFP protein level in the whole-cell lysates of PK-15 cells infected with PRV-GFP with or without SMBS treatment. **(F,G)** Flow cytometry analysis of the percentage of GFP^+^ cells in PRV-infected PK-15 cells treated with the metabolites obtained from *B. subtilis* L2 and V11 at indicated periods **(F)** or at various concentrations **(G)**. Scale bars, 100 μm **(B)**. Data are pooled from three independent experiments (mean + s.d.). ns, not significant; **P < 0.01; ***P < 0.001 (Student’s paired *t*-test).

To know if the secondary metabolites of *B. subtilis* L2 (SMBS-L2) or V11 (SMBS-V11) have antiviral activity, we firstly collected the supernatants of the two strains cultured for 24 h in LB medium. To determine the cytotoxicity of SMBS, we examined the effect of both SMBS-L2 and SMBS-V11 on the cell viability by MTT assay. The results showed that both SMBS-L2 and SMBS-V11 have no obvious cytotoxicity in PK-15 cells at the test concentrations (Figure 2A). Therefore, we next investigated the antiviral effect of SMBS of L2 and V11, respectively, *in vitro*. While SMBS-L2 treatment remarkably suppressed the propagation of PRV-GFP in PK-15 cells, SMBS-V11 treatment has no effect on virus replication (Figure 2B), suggesting that SMBS-L2 contain antiviral components but not that of SMBS-V11. Flow cytometry analysis also showed that SMBS-L2 treatment significantly decreased the proportion of GFP positive cells upon PRV-GFP infection (Figure 2C), further confirming that SMBS-L2 could inhibit PRV replication *in vitro*. In contrast, SMBS-V11 treatment did not impair the propagation of PRV-GFP in PK-15 cells (Figure 2C), which indicated that SMBS-L2 likely contained different components from that of SMBS-V11. In addition, SMBS-L2 treatment also suppressed the propagation of PRV-GFP in both HeLa and BHK-21 cells, indicating that the antiviral activity of SMBS-L2 is not cell-specific (Supplementary Figures 1A, B). Such phenomenon was further verified by qPCR or immunoblotting analysis to show that viral gene copy number of PRV glycoprotein gD (Figure 2D) and GFP protein levels (Figure 2E) were both strikingly reduced in PK-15 cells infected by PRV-GFP upon SMBS-L2 treatment. To know in which stage the antiviral activity of SMBS-L2 was generated, we collected the supernatants at different culture times of *B. subtilis* L2. Although SMBS-L2 collected at the early culture stage had no antiviral activity, the bacterial metabolites obtained after 24 h all showed remarkably antiviral activity against PRV (Figure 2F), indicating that *B. subtilis* L2 produced antiviral metabolites cumulatively. As expected, SMBS-V11 collected at both the early culture stage and late culture stage had no antiviral activity (Figure 2F), which is consistent to the above observation. Since the antiviral activity of SMBS-L2 obtained at different culture times was similar after 24 h, we therefore used SMBS generated at 24 h for subsequent experiments. Not surprisingly, SMBS-L2 treatment strikingly suppressed PRV replication in a dose-dependent manner while SMBS-V11 treatment had no antiviral activity even at a relative high concentration (Figure 2G). Collectively, these results demonstrate that SMBS-L2 contain antiviral components against PRV.

### The antiviral activity of SMBS-L2 is thermal stable and resistant to protease digestion

**FIGURE 3:**
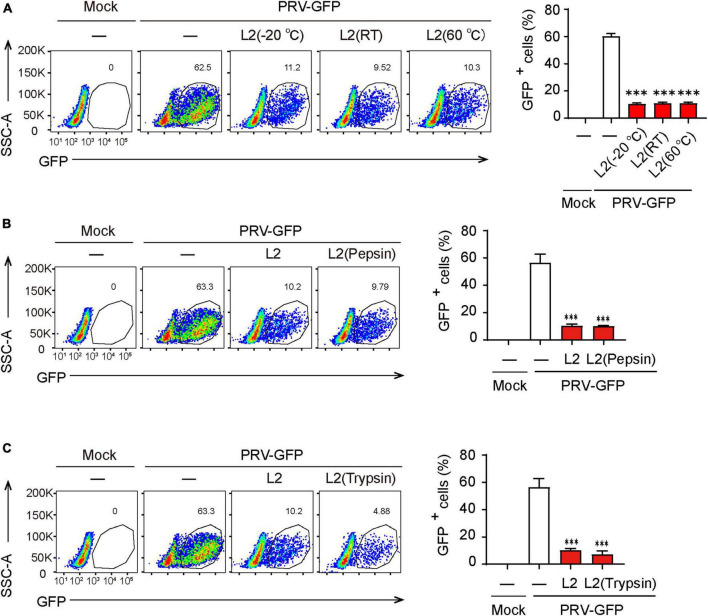
SMBS of L2 contain non-protein antiviral components that are thermal stable. **(A–C)** Flow cytometry analysis of GFP^+^ cells in PRV-infected PK-15 cells following treatment with SMBS that was treated with pepsin or trypsin for 2 h **(A,B)**, or stored at 60°C, 25°C or –20°C for 10 days **(C)**. Data are pooled from three independent experiments (mean + s.d.). ns, not significant; ***P < 0.001 (Student’s paired *t*-test).

Given that the stability of active components in metabolites is critical for its extraction and application, we then determined the thermal stability of the antiviral components in SMBS-L2. Firstly, SMBS-L2 were stored at −20°C, room temperature or 60°C for 10 days, respectively, and then the antiviral activity of SMBS-L2 were further examined. The results showed that SMBS-L2 kept in distinct conditions still had similar antiviral activity against PRV in PK-15 cells (Figure 3A), suggesting that antiviral activity of SMBS-L2 is regardless of the storage temperature. In addition, SMBS-L2 digested by pepsin or trypsin with a final concentration of 100 μg/mL for 2 h still remained antiviral activity (Figures 3B, C), further indicating that the ingredients with antiviral activity in SBMS-L2 are unlikely to be proteins. Taken together, these results demonstrate that SMBS-L2 exhibit good thermal stability and could be a potential candidate for novel antiviral drugs.

### SMBS-L2 inhibit PRV infection at multiple stages of virus life cycle

**FIGURE 4:**
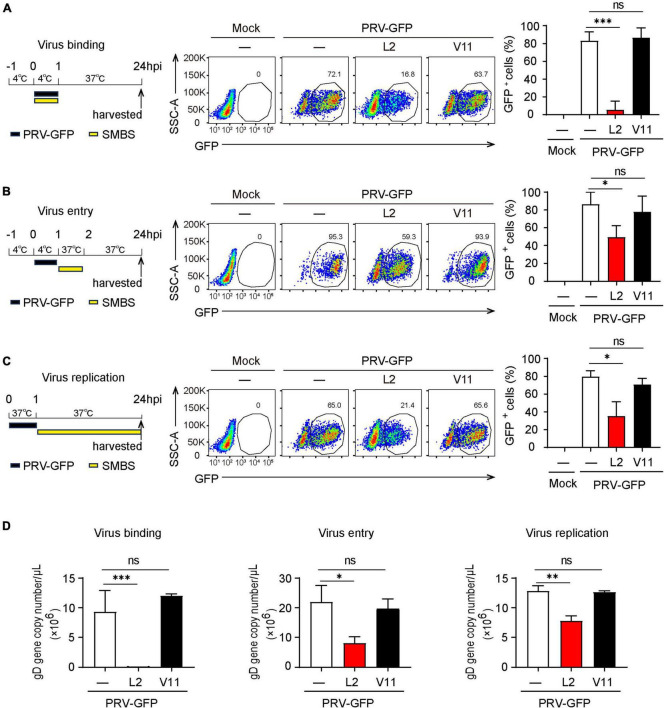
SMBS of L2 inhibits the PRV binding, entry and replication in PK-15 cells. **(A–D)** Flow cytometry of the percentage of GFP^+^ cells **(A–C)** or qPCR analysis of gD gene copy number of PRV **(D)** in PK-15 cells infected with PRV-GFP upon the addition of SMBS at viral binding, entry or replication stages, respectively. Representative data are shown in the left and statistical data (right) are pooled from three independent experiments (mean + s.d.). ns, not significant; *P < 0.05; **P < 0.01; ***P < 0.001 (Student’s paired *t*-test).

Next, we wanted to determine which viral infectious stage that SMBS-L2 likely target to repress PRV infection. Flow cytometry analysis showed that SMBS-L2 strikingly inhibited PRV binding to PK-15 cells and also significantly suppressed the entry of PRV in PK-15 cells (Figures 4A, B), which suggested that SMBS-L2 could function at both viral binding and entry steps to prevent PRV infection. Moreover, SMBS-L2 inhibits PRV binding regardless of its adding order (Supplementary Figures 2A, B) and the replication of PRV was also blocked by SMBS-L2 treatment (Figure 4C). As a result, qPCR analysis also showed that gD gene copies of PRV were reduced at multiple stages of virus infection (Figure 4D). Not surprisingly, SMBS-V11 treatment had no significant antiviral effect at any stages of PRV life cycle (Figures 4A–D). In addition, SMBS-L2 treatment did not alter *IFNB1* expression in PK-15 cells (Supplementary Figure 2C). Altogether, SMBS-L2 possibly target at multiple stages of virus life cycle to prevent PRV infection *in vitro*.

### Oral administration of SMBS-L2 protects mice from PRV infection *in vivo*

**FIGURE 5:**
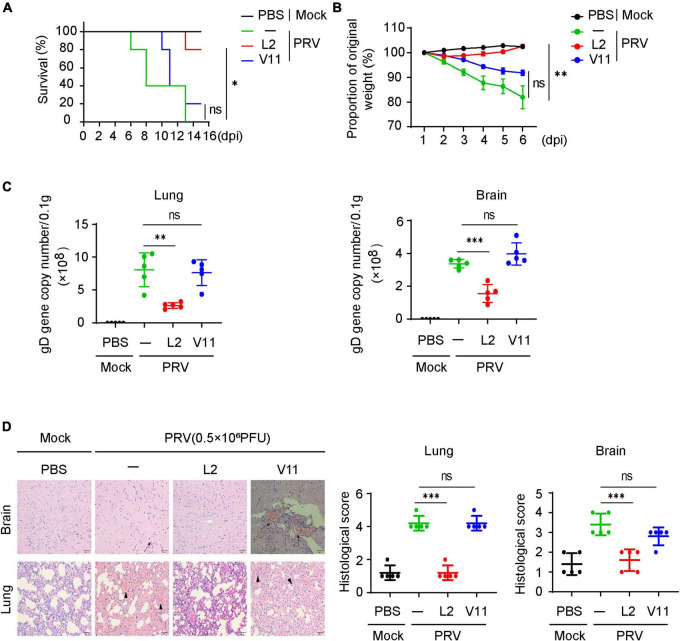
SMBS of L2 treatment suppresses PRV infection *in vivo*. **(A)** Survival rate of C57BL/6 mice (*n* = 5 in each group) infected with a lethal dose (0.5 × 10^6^ PFU, the same below) of PRV (Bartha-K61 strain) pretreated with or without SMBS of L2 or SMBS of V11. **(B)** Body weight changes of mice infected with PRV as in **(A)** for indicated periods. **(C)** qPCR analysis of viral loads in tissue of mice brain and lungs at day 6 after infection with a lethal dose PRV (*n* = 5 in each group). **(D)** Hematoxylin and eosin (H&E) staining and histological scoring of sections of brain and lungs of mice as in **(C)**. Scale bars, 200 μm. Original magnification, ×20. ns, not significant; *P < 0.05; **P < 0.01; ***P < 0.001 [Student’s unpaired *t*-test or long-rank test in **(A)**].

Having known that SMBS-L2 inhibit PRV infection *in vitro*, we then investigated whether SMBS-L2 could have antiviral activity *in vivo*. To know that, mice were infected with a lethal dose of PRV Bartha-K61 following orally administration of SMBS-L2 or SMBS-V11 for continuous 5 days. We found that mice infected PRV led to 100% mortality in mice at 13 dpi, yet SMBS-L2, but not SMBS-V11, administration significantly enhanced survival rate to mice infected with PRV (Figure 5A), which suggested SMBS-L2 did protect mice from PRV infection *in vivo*. Indeed, SMBS-L2 treatment also restored the body weight loss caused by PRV infection (Figure 5B). Additionally, SMBS-L2 treatment remarkably reduced the virus copies in the brain and lung from mice infected with PRV compared to that of mice infected PRV with SMBS-V11 or saline treatment (Figure 5C). Consequently, PRV-infected mice without SMBS-L2 exhibited severe histopathological changes in lung and brain including viral encephalitis characterized by cerebral vascular congestion and hemorrhage in brain, as well as interstitial pneumonia or hemorrhagic pneumonia in lung (Figure 5D) while PRV-infected mice with SMBS-L2 administration exhibited mild histopathological changes. Taken together, these data demonstrated that SMBS-L2 treatment could protect mice from PRV-mediated pathogenicity *in vivo*.

## Discussion

While large-scale production and widespread distribution of vaccines and antiviral drugs, viruses are still causing variety of diseases in humans and animals. Recently, probiotics with antiviral activity or probiotic metabolites are documented as one of the novel therapeutic agents or adjuvants for vaccines in treating viral infections, including *B. subtilis*, a widely applied probiotic in agriculture and industry (Kovacs, 2019; Nayebi et al., 2022; Wang et al., 2022). Nonetheless, the beneficial effects of *B. subtilis* are shown to be strain-dependent, and the strain-specific production of secondary metabolites is likely related to horizontal gene transfer enabled by the natural competence of *B. subtilis* under stress (Garcia-Vallve et al., 2003; Mielich-Suss and Lopez, 2015). Considering the diverse habitats of *B. subtilis*, it is not surprising that certain strains have acquired new biosynthetic gene clusters (van Wezel and McDowall, 2011). Indeed, here we identified two novel *B. subtilis* strains from chicken fecal samples did have distinct features. Particularly, the secondary metabolites of *B. subtilis* L2, but not that of *B. subtilis* V11, exhibited relatively good antiviral activity against PRV both *in vitro* and *in vivo*, highlighting the importance of discovery of novel antiviral drug from secondary metabolites of *B. subtilis*. Interestingly, SMBS-L2 displays no cytotoxic effects on PK-15 cells in tested concentrations and the antiviral components in SMBS-L2 are likely thermal stable and non-protein factors although we still do not know the exact elements with antiviral activity. Hence, our results suggest that the secondary metabolites of *B. subtilis* may be useful resources for isolation and identification of new antiviral compounds. Nevertheless, the specific antiviral molecules within the metabolites and their possible mechanism of antiviral activity are important questions to be answered. In fact, we have purified five major components from SMBS-L2 by thin layer chromatography and 3 of them exhibit antiviral activity against PRV in a dose-dependent manner (Supplementary Figures 3A–E). Further study needs to be performed to identify the exact molecules and elucidate their modes of action responsible for its antiviral activity.

Pseudorabies virus (PRV) belongs to the alphaherpesvirus subfamily of virus, which infects a broad range of vertebrates, particularly swine. PRV infection can lead to severe diseases characterized by respiratory distress, neurological symptoms, and high mortality rates in swine (Hu et al., 2015; Sehl and Teifke, 2020; Andreu et al., 2021). Moreover, recent studies have shown that variant PRV can directly transmit from animals to humans in certain conditions (Fan et al., 2020; Li et al., 2020; Liu et al., 2021). Current antiviral drugs may be toxic to human and animal cells, particularly in case of DNA replication inhibitors or combination therapies due to their impact on host cell replication. Hence, there is a significant need to develop new antiviral drugs that focus more effectively on targeting viral binding and entry, rather than solely concentrating on viral replication (Hall et al., 2011; Pau and George, 2014; Kausar et al., 2021; Menendez-Arias and Delgado, 2022). In fact, several natural products, such as dandelion aqueous extract, glycyrrhiza polysaccharide and huaier polysaccharide, were reported to be capable of impeding viral binding or entry (Cai et al., 2022; Huan et al., 2022a,b). Consistent with the effects of these various antiviral natural products, we found that the metabolites of *B. subtilis* L2 effectively demonstrated antiviral functions against PRV infection at multiple stages of virus life cycle *in vitro*. It is worthy to note that, compared to its effect on viral entry and replication steps, the metabolites of *B. subtilis* L2 have a more pronounced effect on the binding of PRV. This suggests a significant inhibition by the metabolites on the binding of PRV envelope protein gC to the host cell surface heparan sulfate proteoglycan. Indeed, *B. subtilis* has been reported to be capable of inhibiting viral attachment, for example, *B. subtilis* 168 and OKB105 can inhibit the entry of transmissible gastroenteritis virus into the intestinal epithelial cells by competing for the viral-entry receptors (Wang et al., 2017). In addition, the inhibition of PRV entry by the metabolites of *B. subtilis* L2 suggests that these metabolites might also impede the interaction between PRV gD protein and swine nectin-1, thereby hindering the viral DNA from entering the host cell. Although we demonstrated that PRV-infected mice following successive orally administrations were significantly protected by SMBS-L2, it may also prevent PRV infection due to their inhibition on PRV binding and entry.

In summary, we have demonstrated that the secondary metabolites generated by *B. subtilis* L2 effectively prevent PRV infection possibly at multiple stages of virus life cycle. Importantly, these *B. subtilis* L2 metabolites are also capable of controlling PRV infections *in vivo*. These findings not only extend the antimicrobial potential of *B. subtilis* metabolites but also highlight the prospect of isolating novel antiviral compounds from probiotic metabolites. Hence, our research offers innovative insights that could inspire the development of natural metabolites for combating DNA virus infections.

## Data availability statement

The raw data supporting the conclusions of this article will be made available by the authors, without undue reservation.

## Ethics statement

The animal study was approved by the Shandong Agricultural University Animal Care and Use Committee (Approval number: # SDAUA-2018-057) and were conducted in strict accordance with the Animal Ethics Procedures and Guidelines of the People’s Republic of China. The study was conducted in accordance with the local legislation and institutional requirements.

## Author contributions

XW: Data curation, Formal analysis, Investigation, Methodology, Writing – original draft. GH: Data curation, Writing – review and editing. MZ: Writing – review and editing, Investigation, Methodology. MC: Investigation, Writing – review and editing, Formal analysis. HL: Formal analysis, Writing – review and editing, Project administration, Resources. YS: Conceptualization, Project administration, Writing – review and editing, Funding acquisition, Supervision, Formal analysis, Writing – original draft.
