# Supplementary material for: Secondary metabolites of Bacillus subtilis L2 show antiviral activity against pseudorabies virus
Source: Front Microbiol. 2023 Oct 30;14:1277782. doi: 10.3389/fmicb.2023.1277782 (PMC10642297; doi:10.3389/fmicb.2023.1277782)
Supplement: Supplementary file 1 [file Data_Sheet_1.docx]

Supplementary Material


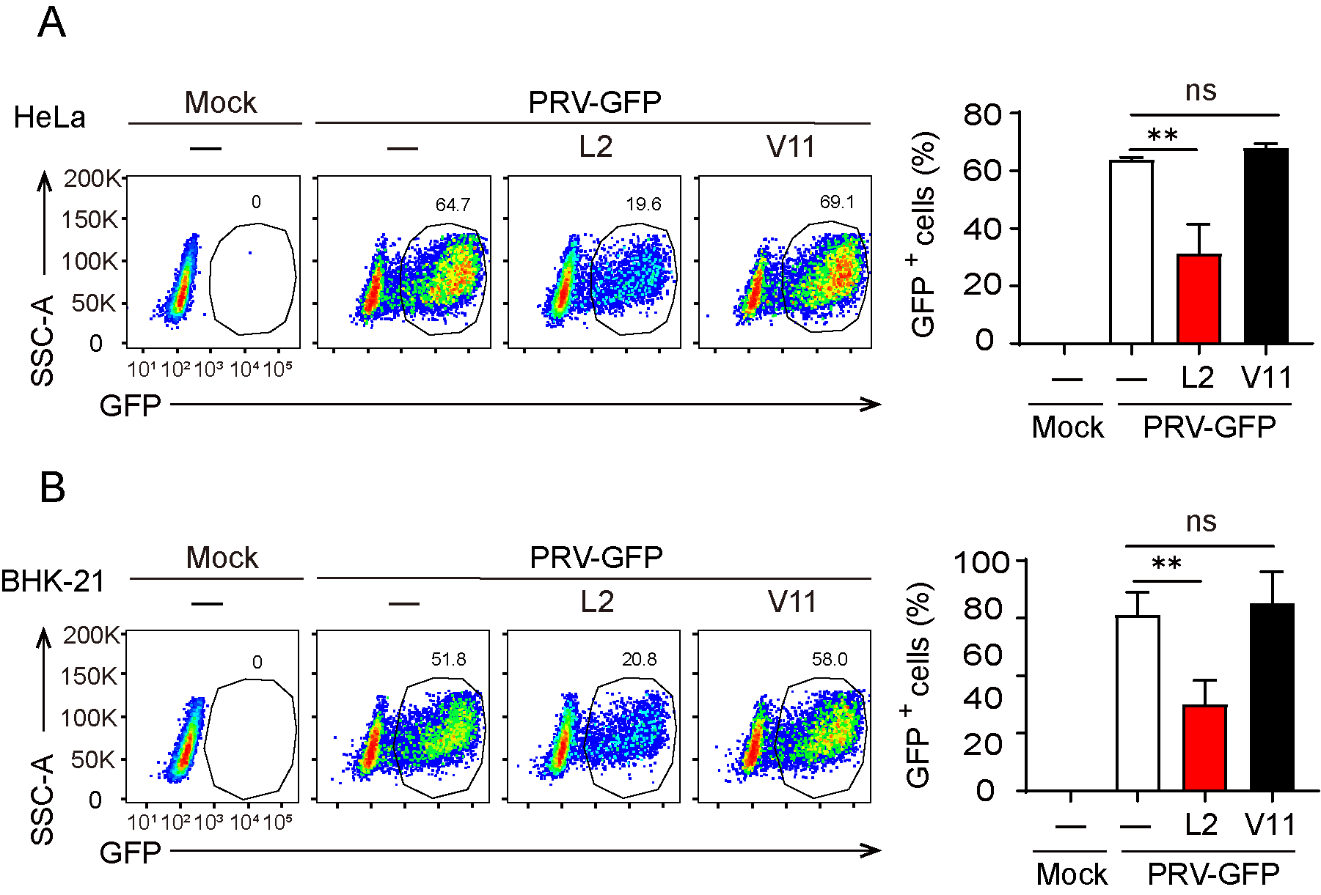


**Supplementary Figure S1. SMBS of L2 significantly inhibits PRV replication in multiple cells *in vitro*. (A, B)** Flow cytometry analysis of HeLa cells (A) or BHK-21 cells (B) left untreated or infected with PRV-GFP (A, MOI=1; B, MOI=0.5) following SMBS treatment. Representative data are shown in the left and statistical data (right) are pooled from three independent experiments (mean + s.d.). ns, not significant; ***P* < 0.01 (Student’s paired *t* test).


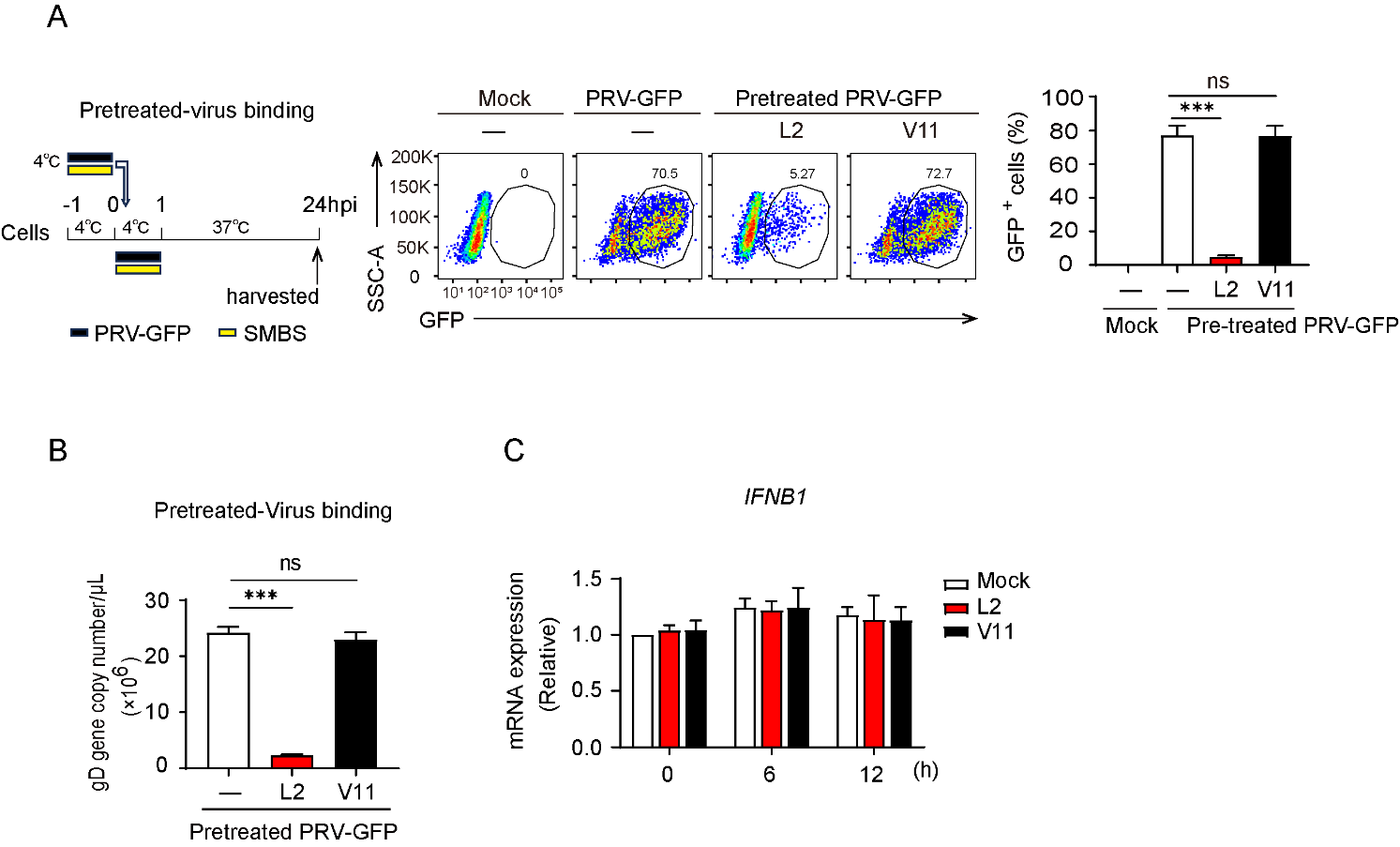


**Supplementary Figure S2. SMBS of L2 inhibits PRV binding regardless of the adding order and without altering IFN expression. (A, B)** Flow cytometry analysis of the percentage of GFP^+^ cells (A) or qPCR analysis of PRV gD gene copy number (B) in PK-15 cells infected with PRV-GFP pretreated with SMBS-L2 for 1 h before adding to cells for virus binding assay. **(C)** qPCR analysis of the *IFNB1* expression in PK-15 cells following treatment with SMBS of L2 or V11 for indicated times. Data are pooled from three independent experiments (mean + s.d.). ns, not significant; ****P* < 0.001 (Student’s paired *t* test).


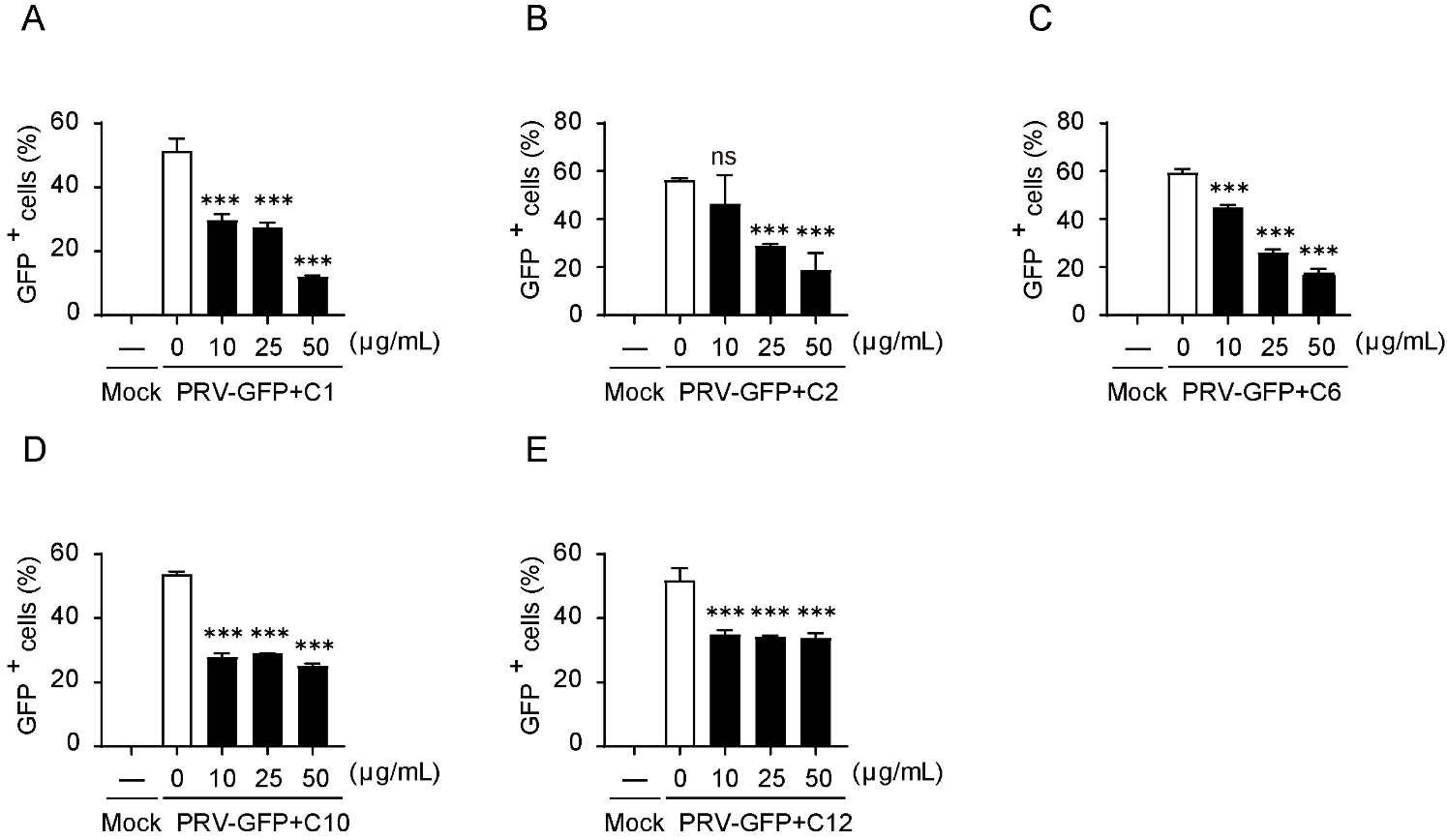


**Supplementary Figure S3. Key purified components from SMBS-L2 show antiviral activity against PRV in a dose-dependent manner.** **(A-E)** Statistics of GFP^+^ cells by flow cytometry in PK-15 cells left untreated or infected with PRV-GFP following treatment with different components (C1, C2, C6, C10, C12) that are purified from SMBS of L2 at indicated dosage. Fractions were extracted with acetone from SMBS-L2 and were fractionated using silica gel column chromatography and thin layer chromatography. Statistical data are pooled from three independent experiments (mean + s.d.). ns, not significant; ****P* < 0.001 (Student’s paired *t* test).
